# Usability evaluation with mental health professionals and young people to develop an Internet-based cognitive-behaviour therapy program for adolescents with anxiety disorders

**DOI:** 10.1186/s12887-015-0534-1

**Published:** 2015-12-16

**Authors:** Lori Wozney, Pamela Baxter, Amanda S. Newton

**Affiliations:** Centre for Research in Family Health, IWK Health Centre, Halifax, Nova Scotia B3K 6R8 Canada; School Of Nursing, McMaster University, Hamilton, Ontario L8S 4 K1 Canada; Department of Pediatrics, Faculty of Medicine & Dentistry, University of Alberta, Edmonton, Alberta T6G 1C9 Canada

**Keywords:** Usability testing, Adolescents, Anxiety, Cognitive behaviour therapy, eHealth, Intervention research, User experience, Mental health, Internet-based

## Abstract

**Background:**

Use of the Internet to deliver cognitive behavioural therapy, a frontline treatment for anxiety disorders, is emerging as an option to increase access to treatment among adolescents with anxiety disorders. This study examined the usability of the Internet-based component of Breathe, a CBT program designed for adolescents with mild to moderate anxiety and impairments.

**Methods:**

A mixed-method usability testing design with semi-structured interviews, task completion, and survey by trained usability moderators was undertaken with two interactive cycles to determine the usability (ease of use, efficiency, errors, and user satisfaction) of the user interface and content areas of the program. Purposeful sampling was used to recruit mental health clinicians with expertise in treating adolescent anxiety disorders and young people aged 15 to 24 years involved. Testing involved using Web-conferencing software that allowed remote participation through personal computers. Two testing cycles involved participants completing structured ‘think aloud’ and ‘cognitive walkthrough’ tasks within the program. At the end of each cycle participants completed a 15-item global usability evaluation survey and were asked a series of open-ended questions. Descriptive and simple content analyses were used to identify and score usability issues for frequency and severity.

**Results:**

Five clinicians and four young people (all < 20 years of age) participated. Most participants described their computer skills as ‘good’ (60 % clinicians, 50 % young people), and attitudes toward Internet-based health care ranged from negative (75 % young people) to positive (60 % clinicians, 25 % young people). Scores from the global usability evaluation after both testing cycles ranged from 3.5 to 5 out of 5 in strong agreement/support of the program in terms of user performance indicators (i.e., learnability, efficiency and number of errors) and user satisfaction. Participants were able to complete all critical tasks with minimal errors. Errors and issues identified during testing were predominantly around enhancements to the visual design and navigational support. Opinions across usability elements did not differ between young people and clinician participants.

**Conclusions:**

A multi-method remote usability approach provided the opportunity to improve the technical interface, therapeutic messaging and user experience of an Internet-based treatment program for adolescent anxiety disorders.

## Background

In their lifetimes, approximately 1 in every 3 adolescents will meet criteria for an anxiety disorder [1]. Few adolescents, however, receive specialized mental health care [2] for an anxiety disorder including the recommended frontline psychological treatment, cognitive behavioural therapy (CBT). Traditionally delivered in-person, barriers to receiving CBT may include social stigma, direct and incidental costs (e.g., time out of school), lack of trained deliverers, and inconvenient service times and locations [3–6]. Adolescents have also been found to be reluctant to seek professional treatment [7] due to strong beliefs that they should cope alone [8], low mental-health literacy [9], and inadequate social or interpersonal skills [10]. Use of the Web to deliver CBT for adolescent anxiety disorders is emerging as an acceptable treatment option to increase access to treatment among those not wishing to seek in-person professional treatment [11], without available treatments or timely access to treatments, or who prefer to treat their anxiety autonomously [12–14]. While this self-managed treatment approach is considered lower-intensity than in-person treatment, many anxious adolescents experience only mild to moderate distress and impairment [1] making this treatment approach appropriate for these young people.

CBT for anxiety problems in adolescence is very effective at reducing symptoms and improving daily functioning [15]; this includes efficacy in adolescents with co-morbidities [16] and across cultural groups [17]. CBT combines systematic exposure to feared situations with skills training to help replace anxious thoughts about feared situations with more adaptive thoughts [18]. These treatment elements are based on the premise that repeated exposure to feared situations results in a desensitization to them, reducing anxiety and avoidant behaviours, and improving functioning. The structured and sequential nature of CBT translates well to computer-based delivery via the Web [19] and initial research has demonstrated feasibility for a variety of CBT interventions for treating anxiety [20–22].

We developed an Internet-based, CBT treatment program (named Breathe) for anxious adolescents with mild to moderate distress and impairment. The program includes online (CBT treatment modules) and Internet- (e-mail support) based features as well as telephone support provided by a trained health care professional (‘Anxiety Expert’). Development of the treatment modules was overseen by research team members with expertise in treating anxiety disorders, and translation of the modules for online delivery was led by research staff at the Centre for Research in Family Health (Halifax, Nova Scotia, Canada) with extensive experience in developing and delivering online interventions [23, 24]. The Breathe program includes the core CBT clinical treatment components outlined by Friedberg and McClure [25] (i.e., mood-check-in, homework review, collaborative goal setting, psycho-education/discovery, planning/homework, and feedback) as 8 interactive modules written at a grade 5 reading level.

Breathe Module Topics:

- Module 1 (Introduction to Breathe/Anxiety Overview)
- Module 2 (Negative Power of Unrealistic Beliefs)
- Module 3 (Common Thinking Traps)
- Module 4 (Relaxation Strategies and Positive Coping)
- Module 5 (Avoid Avoiding/Preparing to Face Fears)
- Module 6 (Fear Ladders/Strategy Planning)
- Module 7 (Practicing Exposure Activities/Dealing With Set-Backs)
- Module 8 (Maintaining Gains)

Treatment modules include the following: (a) video-delivered education about anxiety problems and approaches; (b) safety-monitoring and assessment of different intensities of need and anxiety severity; (c) activities to teach adolescents how to identify anxious thoughts and develop realistic thinking about anxiety-producing situations; (d) coping skill activities with self-assessment and rewards; (e) development of a hierarchy of feared situations and steps for gradual and repeated exposure to feared situations (using imagery and in vivo activities); (f) contingency management; and (g) peer modelling. Automated e-mail support to parents is also an option within the program.

The Breathe program is powered by the Intelligent Research and Intervention Software (IRIS ©) platform that supports persuasive technology elements that make the program more than a static Internet-based treatment on a computer. IRIS provides customization (e.g., using responses to hide/show only relevant content), personalization (e.g., pre-populating worked examples, email reminders, etc. with the user’s demographic information or previous responses), and notifications (both to the user or program staff, within the platform and to outside emails) to ensure the program adapts to the adolescent user to provide a tailored treatment approach.

Internet-based health programs and interventions require a robust and bug-free underlying architecture, and user experience design that aligns with skill and knowledge level [26]. The failure of the interface to meet user demands can result in decreased effectiveness, efficiency, satisfaction and diminished task performance [27]. Usability testing provides critical user feedback about what works and what doesn’t work in the interface, and where there are gaps (technical and aesthetic) that might affect program/intervention performance or user satisfaction [28–30]. For Internet-based health programs and interventions with limited offline support, usability issues need to be closely scrutinized to maximize users’ ability to self-direct through the treatment.

The purpose of the present study was to explore the usability of Internet-based component of the Breathe program in terms of 1) how quickly users were able to learn the new interface and program layout (learnability); 2) the ease with which users could navigate through the components of the intervention (efficiency); 3) the frequency and severity of usability issues and 4) satisfaction with program content and the Website interface.

## Methods

Drawing on lean user experience design (UX) [31] and agile software development approaches (ASD) [32], we followed an iterative and participatory process to evaluate the Breathe program. Usability testing sessions were designed for remote delivery using ‘Elluminate Live!, an online Web-conferencing tool that facilitates real-time interactive discussions, polling, shared desktop and session recording features. This approach is a reliable method for conducting evaluations of Websites [33] and removed geography as a barrier to study participation. The study protocol was approved by the Health Research Ethics Boards at the University of Alberta (Edmonton, Alberta, Canada) and IWK Health Centre (Halifax, Nova Scotia, Canada).

### Participant selection

A purposive sample of mental health clinicians with expertise in treating adolescent anxiety disorders and young people aged 15 to 24 years involved in peer advocacy for mental health and illness were recruited. There were no exclusion criteria for youth as usability testers; youth could self-identify for study participation out of interest based on personal (i.e., personal experience with anxiety) or professional (i.e., peer advocacy activities) reasons.

Clinicians and young people were recruited through a national contact (e-mail) list maintained by the National Infant, Child and Youth Mental Health Consortium in Canada. Individuals on this list volunteered their contact information to the Consortium through participation in national mental health related activities and involvement in advocacy and education groups for mental health. All individuals on the contact list were e-mailed a study information letter inviting them to participate. This method was supplemented by convenience sampling of clinicians contacted during a Canadian Mental Health Association (CMHA) webinar hosted by the research team and youth volunteers working on mental health related projects at the Centre for Research in Family Health.

We employed a broad age range for recruiting young people that is consistent with the World Health Organization and United Nations age-range for “young people” [34]. While the program is designed for adolescents, we felt at the usability stage that young people slightly older than the program’s target population could provide valuable input based on past, and relatively immediate, experiences. All participants needed to be able to read and write English and have access to a computer with Internet connection. Youth participants were given a $50 gift certificate as an honorarium for participation. Parental consent was not required. As recruited youth were 15–24 years of age and involved in peer advocacy for mental health they were expected to have advanced knowledge and understanding of the nature of the proposed research, the anticipated risks and potential benefits, and the requirements of the research to be able to make their own, informed decision on whether to voluntarily participate. Youth were emailed an informed consent document that consisted of an information sheet and consent form. They were advised that subsequent participation in the study usability testing session was based on overt action; meaning if they attended the session, they were consenting to the study but could withdraw at any time.

### Study design and procedures

All study participants gave informed consent over the phone/email to the research assistant and were given the opportunity to ask any questions. Participants were mailed headsets with voice-cancelling microphones in advance if needed in order to ensure high quality audio experience during testing and to optimize data quality for transcription. An e-mail notice was sent out 2 weeks prior to the scheduled testing session, providing a Web link to a test session so that participants could ensure their computer had the necessary JavaScript updates and to test their audio set-up.

Prior to starting the session all participants verbally confirmed informed consent. Participants were then asked to complete a general information questionnaire on demographic characteristics and level of comfort with computers and the Internet. Participants then logged into the Breathe program site and participated in standardized ‘think aloud’ [35] and ‘cognitive walkthrough’ [36] activities. Participants were guided through various critical tasks in different modules (e.g., login, complete check-in, view videos, attempts to generate homework activities). All participants worked through the same set of tasks and program components but the tasks were different in each cycle. Cycle 1 testing focused on program modules 1-4 and Cycle 2 on program modules 5–8 though some core features of the program (i.e., check-in, check-out, safety screening, homework pages) were in all modules so there was some overlap. Participants responded to semi-structured interview questions at the end of each session. Testing sessions were digitally recorded and transcribed verbatim by an experienced transcriptionist. To ensure anonymity, all participants were identified by order in which sessions were conducted (participant = Participant 1) and by participant type (young person = Y; clinician = C).

Two session moderators co-led each testing session. These moderators were skilled in qualitative interviewing and trained in using the Elluminate Live! software. Moderators made detailed notes during each session and created summary reports based on their observations. After each testing cycle, the research team and IRIS software programmers identified and prioritized usability issues and solutions.

### Measures

#### Demographic and computer skill survey

Participants were asked by usability testing moderators to rate their own comfort level with computers from 1 ‘very bad’ to 5 ‘very good’ and provide information on how frequently they used computers for various purposes on a scale of 1 ‘never’ to 4 ‘daily’. Additional questions identified attitudes toward Internet-based health interventions, preferred Websites for accessing health-related content and demographic characteristics.

### Program content and usability questionnaire

This 15-item self-report measure collected participant evaluations of Breathe on a likert scale ranging from 1 ‘strongly disagree’ to 5 ‘strongly agree’. Items were adapted from System Usability Scale (SUS) [37] and the Standardized User Experience Percentile Rank Questionnaire (SUPR-Q) [38]. Participants were told that the purpose of the questionnaire was to provide feedback on how user-friendly the program was and which aspects of the program needed the most revision. Participants completed the usability questionnaire at the end of Cycle 1 and Cycle 2 testing sessions.

### Semi-structured interview

A semi-structured interview was used to explore user satisfaction and interface performance. The content of the questions included questions related to 1) usability as a feature in treatment adherence (e.g., What kinds of challenges might youth face when engaging with these on-line modules?); 2) how motivation influences impressions of usability (e.g., What do you think are the benefits or rewards for engaging with Breathe modules?); and 3) context issues for usability (e.g., What would be the best way for youth to use Breathe: alone, with a counsellor, or in a group?). A moderator used probes to elicit specific examples (e.g., Can you tell me more about that?). Participants were asked for suggestions or alternatives and given the opportunity to provide any other relevant comments about the Breathe program.

### Data analysis

Questionnaire data were analysed to determine measures of central tendency and distribution of values. As each cycle of testing focused on different module components of the program, repeated measures analysis was not appropriate. Simple content analysis was performed after each testing cycle on the transcribed data relating to the think aloud and cognitive walk-though activities, and semi-structured interviews. Consideration was given to both positive and negative feedback. Usability researchers commonly rely on the frequency of errors to prioritize changes; however, there is little evidence of the correlation between frequency and severity of usability issues [39]. For this reason content analysis was used to identify key themes (based on frequency) as well as determine error severity level using common severity metrics used in the field of usability testing [40, 41]. Severity levels were defined as: 1 = cosmetic/minor (i.e., subtle and possible enhance enhancements/suggestions); 2 = moderate (i.e., user will be able to use the intervention but will have to undertake some moderate effort in getting around the problem); and 3 = critical/severe (e.g., create significant delay or frustration or prevents a task from being completed). The same analysis process was used for both testing cycles.

## Results

### Participant characteristics

Nine participants (young people, *n* = 4; child mental health clinicians, *n* = 5) participated in Cycle 1 and eight of that original group participated in Cycle 2 (young people, *n* = 4; child mental health clinicians = 4). Usability sessions were conducted between June 27th and July 24^th^, 2014 (Cycle 1) and between September 4^th^ and 10^th^, 2014 (Cycle 2). On average, usability testing sessions took 133 min to complete.

Place of geographic residence spanned Canada for both youth (Western Canada, *n* = 3; Eastern/Atlantic Canada, *n* = 1) and clinician (Western Canada, *n* = 3; Eastern/Atlantic Canada, *n* = 2) participants. The clinician group included three clinician scientists/professors, a social worker and a family therapy coach, all with advanced university education. Clinicians ranged in age from 20–40 years (*n* = 1) to > 40 years (*n* = 4) with both males (*n* = 2) and females (*n* = 3) participating. Young people were all younger than 20 years of age. Both males (*n* = 2) and females (*n* = 2) participated. Information on participant technology use is presented in Table 1. All of the participants reported access to a home computer and reported having at least “average” computer skills or better. All participants reported daily use of computers, Internet browsing and email. Youth testers were not high users of video games or video chat but did report using the computer more often than clinicians. Before Cycle 1 testing, 3 of the 5 young people reported a negative attitude toward Internet-based health care and preference for accessing mental health resources from a health care professional.

**Table 1:** Information on technology use by study participants

	Young people (*n* = 4)	Clinicians (*n* = 5)
	*n* (%)	*n* (%)
Computer at home		
Yes	4 (100)	5 (100)
No	0	0
Hours spent on computer each week		
< less than 2	0	2 (40)
3-5	3 (75)	1 (20)
6-10	1 (25)	2 (40)
Online comfort		
Bad	0	1 (20)
Average	3 (75)	2 (40)
Good	1 (25)	2 (40)
Computer skills		
Average	1 (25)	1 (20)
Good	2 (50)	3 (60)
Very Good	1 (25)	1 (20)
Attitude toward online healthcare		
Negative	3 (75)	0
Neutral	0	2 (40)
Positive	1 (25)	3 (60)
Preference for learning about mental health resources		
Internet/Web Search	1 (25)	3 (60)
Health care professional	3 (75)	2 (40)
E-mail use		
Daily	4 (100)	5 (100)
Weekly	0	0
Monthly	0	0
Never	0	0
Online music use		
Daily	1 (25)	1 (20)
Weekly	2 (50)	3 (60)
Monthly	1 (25)	1 (20)
Never	0	0
Online video viewing		
Daily	2 (50)	0
Weekly	1 (25)	2 (40)
Monthly	0	2 (40)
Never	1 (25)	1 (20)
Online game playing		
Daily	0	1 (20)
Weekly	1 (25)	0
Monthly	0	0
Never	3 (75)	4 (80)
Online video chats		
Daily	0	0
Weekly	1 (25)	2 (40)
Monthly	2 (50)	1 (20)
Never	1 (25)	2 (40)

### Learnability

Responses to the Program Content and Usability Questionnaire were positive across testing cycles and user groups. A summary of questionnaire responses is provided in Table 2. Participants felt they could easily and efficiently navigate the intervention (item #2), that the site was user friendly (item # 10), the menu bar was easy to use (item # 5), and that buttons and menu options worked as they expected them to (item #7). Although participants liked the length of the modules (item # 6) the amount of information on each page was consistently scored lower across both cycles and user groups (item #4).

**Table 2:** Global usability evaluations by young people and clinicians in Cycles 1 and 2

	Young people		Clinicians	
	Cycle 1 (*n* = 4)	Cycle 2 (*n* = 4)	Cycle 1 (*n* = 5)	Cycle 2 (*n* = 4)
	Mean (SD)	Mean (SD)	Mean (SD)	Mean (SD)
1. It is easy to use.	4.75 (0.50)	4.50 (0.58)	4.60 (0.55)	4.25 (0.50)
2. It is easy to navigate.	4.25 (0.50)	4.75 (0.50)	4.00 (0.71)	4.50 (0.58)
3. The terms are clear and easy to understand.	4.25 (0.50)	4.75 (0.50)	4.00 (0.71)	4.50 (0.58)
4. Each page (screen) has the right amount of information.	3.50 (0.57)	3.75 (0.50)	3.80 (1.30)	3.75 (0.96)
5. Modules took an appropriate time to complete.	4.25 (0.95)	4.25 (0.96)	3.80 (1.09)	4.25 (0.50)
6. It is easy to use the menu bar.	4.50 (0.58)	4.75 (0.50)	4.60 (0.55)	4.25 (0.96)
7. The buttons and menu options work as I expected them to.	4.25 (0.50)	3.75 (0.50)	4.20 (0.45)	4.50 (0.58)
8. The site is visually pleasing to me.	3.75 (0.50)	4.00 (0.82)	4.40 (0.55)	5.00 (0)
9. It is always clear what to do next.	3.50 (1.00)	4.25 (0.50)	3.00 (1.00)	3.50 (0.58)
10. The site is user-friendly.	4.25 (0.50)	4.75 (0.50)	4.40 (0.55)	4.75 (0.50)
11. Each page (screen) loaded quickly.	4.50 (0.58)	4.00 (1.15)	4.20 (1.79)	4.00 (2.00)
12. The video segments loaded quickly.	4.50 (0.58)	4.00 (1.41)	4.20 (1.79)	3.25 (2.06)
13. I would return to this Website.	4.00 (0.82)	4.00 (0)	4.80 (0.45)	4.50 (0.58)
14. The tone of the material is sensitive for youth seeking help for anxiety.	4.00 (0.82)	3.75 (0.50)	4.40 (0.80)	4.75 (0.50)
15. The tone of the material is appropriate for youth aged 13-17.	4.25 (0.50)	4.00 (0)	4.60 (0.55)	4.75 (0.5)

Open-ended interview responses from clinician participants frequently noted the importance of being explicit with young people during program use about task expectations and preparing them in advance for what next steps would be. Based on suggestions from young people and clinicians several changes were implemented to improve navigation and learnability 1) a short virtual tour of all the menu options and site features was recorded and users were encouraged to watch the video before beginning Module 1; 2) wording was changed to provide more explicit instructions on next steps and what actions were required by the user; 3) more internal links were added to help users navigate between pages of the intervention (e.g., hyperlinks to head back to homepage, hyperlink from their homework page back to their workbench of practice activities). Minor changes to page layouts, reduction of redundant or repetitive language, adding or removing page breaks and making more use of bulleted lists and pop-ups were minor solutions put in place to make the workflow more efficient without sacrificing important therapeutic messages.

In structured CBT-based modules where content is delivered with a certain chronological logic (e.g., practice low-intensity exposure activities, review, plan for high-level exposure activities, practice moderate-level intensity exposure, etc.) there is a structure to the treatment that require certain elements remain ‘unopened’ until other elements have been completed. During Cycle 1 most of the young people commented on automated restrictions around which content they could access, in which order, how much control they had over choices (e.g., they could not skip from Module 1 to Module 4). Following Cycle 1, an animated video introduction CBT approaches was created and reviewed with users during Cycle 2 testing. Both youth and clinician testers commented that this introductory video would help program users manage their expectations about what would be involved in treatment, how long it would take and why CBT sessions were structured sequentially. Additional, positive language was added to the text in exposure focused Modules (5–7) to help users understand why a stepped approach was being used and to encourage them to continue. A frequently cited benefit of the intervention in terms of learnability and ease of use was automated email notifications for youth that would offer encouragement and reminders for completing activities if you had not logged onto the system or completed a module after a given amount of time.

### Technical errors and efficiency

The majority of usability issues identified in both cycles were identified as Level 1, cosmetic or minor (see Table 3) and included things like 1) color and palette issues, typos, legibility problems and alignment or layout flaws. All users were able to perform critical testing tasks (e.g., login, submit check-in, submit homework, access workbench) during both testing cycles. Level 1 issues were most often described by users not as “errors” but ways the site could be enhanced or improved. Moderate errors referred to instances where users were slowed down in completing tasks by; 1) lack of explicit instruction, 2) lack of confirmation that an action had been taken, and 3) inability to navigate within the site. Users could often trial and error their way around these issues and still complete the task, but not efficiently. The bulk of redesign efforts by programmers were spent on addressing moderate errors. Critical errors that negatively affected task completion for non-critical tasks all related to page loading issues, links not redirecting and pop up text not appearing as intended) and were easy for programmers to remedy. Bandwidth issues related to the remote testing software (Elluminate Live!) was a significant mitigating factor for several participants, affecting how quickly pages and videos loaded on the shared screen. This was evidenced in the variability of scores for page and video loading measures (see Table 2, items #11 and #12) but was not an issue with the Breathe site itself.

**Table 3:** Severity level of usability issues identified in Cycle 1 and Cycle 2

	Youth		Clinicians	
	Cycle 1 (*n* = 4)	Cycle 2 (*n* = 4)	Cycle 1 (*n* = 5)	Cycle 2 (*n* = 4)
Level 1 (cosmetic/minor)	66	49	117	66
Level 2 (moderate)	22	11	38	14
Level 3 (critical/fatal)	4	5	9	3

### Satisfaction

As the IRIS platform that supports the Breathe intervention allows for a highly dynamic experience for users, it was not surprising that satisfaction was defined and described by users in multiple ways: site aesthetics; relevant content, interactivity, self-direction, and trust.

### Site aesthetics

Consistent with other research in the field, site aesthetics was identified by both test groups as critical to overall usability and satisfaction [42, 43]. Both user groups generally found the site to be user-friendly and visually pleasing (Table 2, item #8). During Cycle 1 most users commented on areas where the content might be “chunked” into smaller amounts of text and more visual assets (i.e., graphics, images, videos) included. All participants commented positively about the quality and age-appropriateness of videos, images, animations, logo, and “comic book” style illustrations but suggested adding more visual cues for key concepts. The desire for additional visual assets was the most frequently cited usability issue for both cycles of testing, with young people proposing more aesthetic enhancements than clinicians. Table 4 provides examples of both positive and negative comments made in relation to site aesthetics. Examples of how the research team addressed issues raised by testers are also provided.

**Table 4:** Examples of feedback on aesthetic, content and learnability elements of Breathe

Elements	Negative feedback	Positive feedback	Changes applied
Layout	“It took me a couple of questions to realize that I wasn’t answering the question below. So that may be something you just want to either get rid of the white spacing or insert the spacing after the answer.” (Y01) “I think there’s almost too much information on this one page… Maybe if that page was broken down into three or four other pages, like that section right there being one page.” (C06)	Yes I’m very impressed with the layout of this… it’s concise and the information is good… I like how there are pictures and if you click the picture there’s lot of info. I found that really cool… it was quick and easy.” (Y03) “Yes, it was very good…kind of leaving some room for just self-directed user discovery which is always kind of fun, especially with sites designed intuitively like the Breathe site is. The layout is very simple and minimalistic, and I can definitely kind of see persistence, kind of enjoying the experience of learning how to use the site.” (Y02)	Converted larger chunks of text into bulleted lists. Limited amount of scrolling users would do. Broke long sections into multiple pages. Created more open text boxes and ‘other’ category for users to express ideas Allowed users to go back over previously completed pages more easily
Audio/Visual	“I also don’t know if you want to stick any images in here because from just my immediate viewing of the page… it just appears as a giant block of text, you may want something to break it up a little.” (Y01) “The voice I found a little bit on the quiet side. It was kind of that background quiet soft hiss… I wondered if the background voice could be louder, or the person speaking, if that could be louder and more crisp?” (C07)	“I kind of like the fact that you recycled the videos of raw images… because it gives a sense of continuity in that each situation can be approached from different angles.” (Y01) “I think [the video] was really interesting because it introduced you to a real person going through something that eventually other youth are going through.” (Y03)	Rerecorded and/or slowed audio recordings for videos. Added visual assets (i.e., photos, graphics, icons and diagrams) to break up page layout.
User-friendliness	“So I can actually print off this page? Okay, because I’m not sure what to do, now that I have finished this log, I’m not sure what I’m supposed to do.” (C08) “There’s just a lot of different things that could be under, what did you do, and I don’t think you can cover all of them, but maybe try to balance them a little bit more, putting in another box so they could type it in.” (Y04)	“One thing I liked was things to try out. I like that it gives you a choice between the two because it’s not always fun to visit one or two things, I like having the choice between the two.” (Y02) “No, nothing looks confusing. Everything looks pretty clear, and maybe that’s why I read the one message and then jumped right in sort of thing. Because it looked pretty easy to use.” (C06)	Modified the tutorial video to provide overview of steps in each module. Changed words on some of the buttons (‘submit’ changed to ‘save’) Drop down menus were added for self-evaluation tasks and open-ended text boxes for adding additional information.
Completeness	“Just a question about this. This might be too complicated, but we have 9-1-1, but is there an ability to link to local hospitals or numbers?” (C07) “I know certainly from personal experiences…it was hard for me to get started, simply in the fact that I didn’t see results immediately. I think you’ve certainly done a good job, but maybe if you say that they actually have to insert some sort of progress report before they receive the next module.” (Y01)	“The thing that impresses me is how comprehensive it is. It’s touching all the bases, so I think that’s a huge advantage. And it’s kind of laid out in a systematic way, so they can do this kind of progressive work through it. And I would think with each module they’re going to feel some benefit, and that’s going to reinforce their motivation to continue.” (C05) “I think it’s nice and brief and to the point, and I do like how it’s showing what you answered the week before. I think it helps put things into perspective… if their moods fluctuate day by day and they are not thinking about success that they had the past week.” (C09)	Created lists of mental health organizations and providers to be filtered by geographic location of user. Provided options for completing extra optional practice activities. Created a Progress Summary graphic to appear at the check-in section of each module.
Relevance	“it seems kind of geared toward youth who are at the start of looking at their anxiety or learning about it, not so much maybe for someone who’s been in counselling for a while or whose already learned a lot about that stuff.” (Y04) “you have to compete with everything else that’s on the Internet and on TV and that’s tough, in order to hold their interest… you almost have to disguise the information that you’re giving them, or the help that you are trying to offer.” (C06)	“It was much more engaging simply because of the use of your images, of the text boxes, of the actual questions. The actual situations you’ve put up are very relatable.” (Y01) “The video is excellent. That’s the comment. That’s what I literally do with teenagers who come in. Yes, and showing it from the side is key because they don’t always understand that. It’s an excellent technique and the explanation is really good too.” (C07)	Rerecorded audio with different voices (e.g., boys and girls speaking). Made some activities optional or fewer “required” activities to be completed Layered some content into image maps, pop down text boxes etc. so youth could decide if they wanted/needed more information
Credibility	“I’m not sure if this would be possible to attach the University of Alberta’s name to the Website or whatever, department or faculty you guys and your research team is based out of…to really communicate to youth that this isn’t just some little thing that just some random person put together somewhere. It is something that people have put a lot of thought and planning and work into, that it’s been evaluated and tested will really help.” (Y02) “I’m thinking about the introductory video… I think I’d be a tad more explicit that there’s lots of documentation that these interventions, these principles are highly effective, so we can say with confidence that you should indeed make progress… and it’s really a function of how much effort you put into this.” (C05)	“I think it’s good that you emphasize the confidentiality of it all. I feel like with the confidentiality they can really express what they’re thinking.” (Y03) “yeah they’re getting *real* < <emphasis> > answers. I just know there’s a lot of ask the experts sort of things where it’s blah, blah, blah, blah, a little blurb, follow-up with your family doctor, blah, blah, blah…”(C09)	Added “Our Team” page to the site, including photos, bios and logos from all participating institutions) Created a tutorial video about the main features of CBT and research evidence of its effectiveness.

### Intervention content

Overall, both young people and clinicians were positive about the age-appropriateness of the program content (Table 2, item #15); its relevance for use by anxious adolescents (Table 2, item #14) and the length of each module (Table 2, item # 6). Clinician responses to interview questions confirmed that, from a therapeutic standpoint, the program included key CBT skills and adequate therapeutic support when needed. Recommendations made by clinicians focused predominantly on risk-endorsement protocols and the proper messaging for adolescents around substance abuse, self-harm and suicidal ideation. As clinicians were not viewing the staff side of the intervention they often wanted to confirm when and how the Anxiety Expert providing support during program use would be notified of user responses. Both young people and clinicians felt that the program would be helpful for adolescents with mild-moderate levels of anxiety, and liked that the program provided a scaffold for users to translate information into behaviour change (progressive exposure to their feared situations) as opposed to just providing information about anxiety.

### Interactivity and self-direction

Participants were positive about the adaptive tailoring features of the IRIS and suggested that this was a critical feature for keeping young people engaged in the intervention over time. While automated personalization was seen as a strength, youth testers also provided feedback about their desire for control over aspects of program workflow (e.g., wanting to decide for themselves if parents should be notified of intervention progress; ability to see a summary of their progress over time, ability to self-select when and how often reminder emails were sent to them). The most common suggestions for additional personalization included: 1) additional space for expression (e.g., places to write out comments, having an ‘other’ option included in a list), and 2) additional choices (e.g., less yes/no options or required answers). Additional opportunity for open-ended comments and more opportunity for program users to select from among a range of options were added. Being designed as a largely self-directed intervention, young people’s responses to questions around ease of use (Table 2, item#1) and ease of navigation (Table 2, item # 2) were especially promising indicators of how easily they could navigate the program on their own.

Clinicians commented several times about legal and regulatory issues around parental involvement in the program (i.e., when parents HAD to be notified of issues, age of consent, protection under Freedom of Information and Protection of Privacy (FOIPOP), etc.). While still acknowledging that young people needed a certain degree of autonomy in the intervention, clinicians were concerned about formal rules around privacy. Young people’s comments tended to focus more on the practice of privacy, of deciding how much information parents received and when. Young people were very positive about how the intervention allowed them more freedom to be honest, to work at their own pace and in their own time. Youth did not show the same concern about online security of their information and did clinicians.

### Trust and credibility

Prior to conducting the usability testing, three of the four (75 %) young people participants indicated they generally held negative attitudes about Internet-based health care and preferred to access mental health resources from their health care provider. During testing sessions young people in particular emphasized concerns about online privacy and credibility. During Cycle 1 testing both young people and clinicians suggested a more transparent/personalized view of the study team (e.g., photos, credentials prominently displayed) and evidence of the program’s credibility/usefulness (e.g., testimonials, endorsements, overview of CBT approaches). Access to the Anxiety Expert via a messaging feature, personal phone follow-up and secure e-mail interaction was frequently noted by test users as a benefit of the program and something that provided a sense of credibility and trust. Two young people commented on how just knowing that “someone” was there was comforting and reassuring even if they never needed to access that part of the intervention. To further enhance this program feature we created an “Our Team” page on the site, which included cartoon portraits of team members along with biographies, and more prominent display of partnering institution logos. The “Ask an Anxiety Expert” feature was also described in the virtual tour video so users would be aware that this feature was available to them during program use.

## Discussion

The iterative approach used in this study enabled us to determine that our Internet-based CBT program was easy to use and understand, was efficient with relevant content, and provided satisfactory user experience. Participants generated innovative suggestions on ways that the Breathe program’s user interface and content could be improved. Although recruitment of youth for health research is frequently cited as challenging [44] our study results suggest that engagement with young people during early phases of intervention development is critical for tailoring online therapeutic interventions to what young people think works and why.

This study highlights the value of conducting formal usability testing to ensure new Internet-based treatments to deliver evidence-based care are usable and developmentally appropriate for the desired population. Our usability testing results included differences in how clinicians and youth may approach Internet-based interventions—with different kinds of scepticism or apprehension. Future research could examine the implementation and decision-making processes used by different stakeholder groups for using online therapeutic interventions. Clinicians in our study focused more on legalities and mechanisms of risk-management in an online environment whereas youth focused more on transparency and personal autonomy (e.g., who was providing oversight, whether parents were involved).

Given that adolescents are more confident technology users and are increasingly accessing information online [45] it was not surprising that the young people in this study found the program easy to use. The young people’s satisfaction with the Breathe program and willingness to revisit the Website again was in contrast to their initial endorsement of negative attitudes towards online health care and preferences for accessing mental health information directly from health care professionals. Although this study involved only a small sample, the role of negative expectations or negative prior online experiences are important potentially mediating factors to explore in future research [46]. All youth participants made multiple comments related to credibility or privacy issues and their impact on ‘buy-in’ for using the intervention. While young people in our study voiced satisfaction with the safeguards (e.g., password protected login, optional parent involvement) and credibility of the Breathe program team, they advocated for increased transparency and explicit information on who was providing oversight. This suggests that if credibility issues are not sufficiently addressed, potential users may not commit to the program, regardless of how engaging and interactive the content may be [47]. Clinicians integrating Internet-based technologies as adjunct to face-to-face treatment with youth may benefit from discussing security/data and credibility concerns with youth up-front and exploring individual preferences for technology-based support. This may be especially true for anxious youth who often face additional challenges in developing computer-mediated therapeutic alliance [48]. Although recent research has examined age related differences in assessing the credibility of Internet-based programs [49, 50], the mechanisms of how perceptions of credibility affect program engagement are still not understood. Clinicians in our study focused less on this aspect of the program compared to young people which suggests that assumptions about young people’s enthusiasm for or preferences related to Internet-based health interventions should be tested empirically [51].

Our findings extend the body of research on usability of Internet-based mental health interventions to show that remote usability testing can produce valuable data and allow for testing with a geographically diverse group. Our study demonstrates that young people can be enthusiastic test users but comparative usability research on age-related difference in testing techniques (e.g., talk aloud, cognitive walkthrough) and testing modality (e.g., remote online versus face-to-face laboratory) is still needed [52, 53]. In our study, young people were eager and able to identify and articulate aesthetic, content and functionality problems while also providing specific suggestions for developers on how to solve problems or enhance the program.

This study was limited by sample size. A more diverse sample (age, geography: rural versus urban residence, technology use) would have allowed us to examine whether personal or experiential factors (motivational attributes, computer confidence, computer expertise) were related to satisfaction. In keeping with usability testing recommendations, our sample was sufficient to identify the large majority of usability issues [54]. It has been estimated that a single usability testing cycle with 5 to 10 users can lead to as much as a 10-fold reduction in issues [55]. By completing two usability cycles, even with a small sample size, we ensured a significant overall reduction in program usability errors prior to feasibility testing.

## Conclusions

This usability study provides evidence for the learnability, efficiency and reliability of a Internet-based CBT program for anxious young people. Iterative testing provided crucial technical and design feedback needed to improve young people’s experience with the intervention. Testing also provided important insights into interactions between context, content and user; particularly how expectations may affect subjective usability results. Breathe is now being evaluated in a pilot randomized controlled trial where issues of feasibility and usability can be further explored with a larger group of youth participants.

## Abbreviations

(ASD): Agile software development
(CBT): Cognitive behavioural therapy
(CMHA): Canadian Mental Health Association
(FOIPOP): Freedom of Information and Protection of Privacy
(IRIS): Intelligent Research and Intervention Software
(SPSS): Statistical Package for the Social Sciences
(SUPR-Q): Standardized User Experience Percentile Rank Questionnaire
(SUS): System Usability Scale
(UX): User experience design
